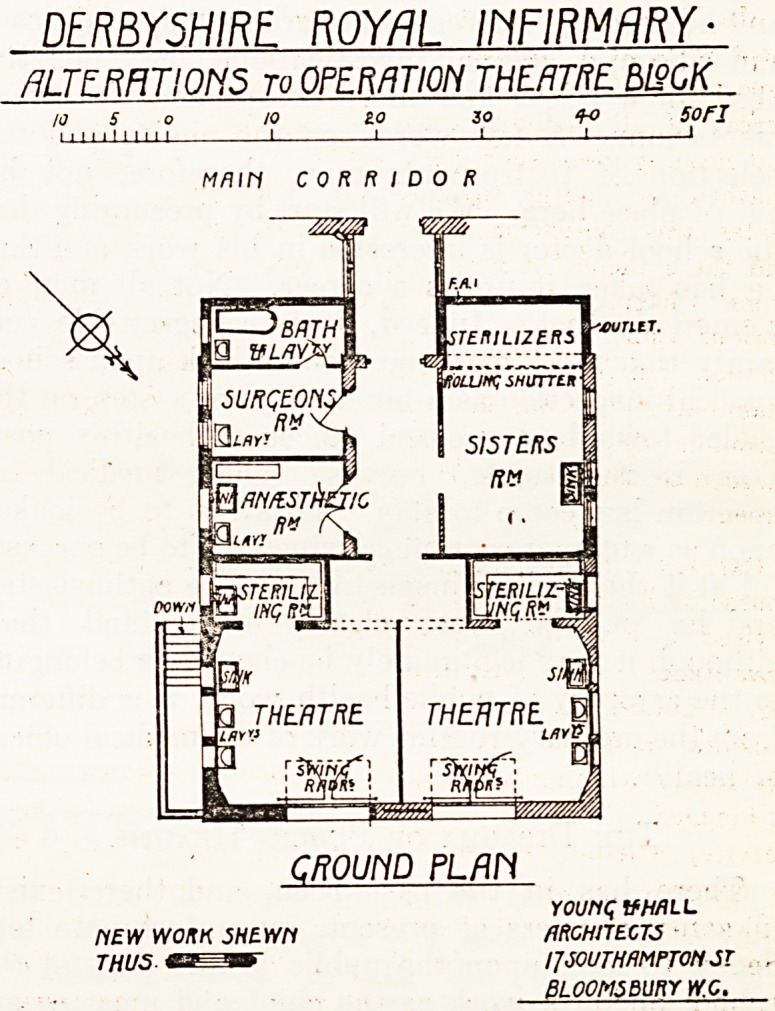# Derbyshire Royal Infirmary

**Published:** 1915-07-31

**Authors:** 


### HOSPITAL ARCHITECTURE AND CONSTRUCTION.

### Derbyshire Royal Infirmary.

### THE ALTERATIONS TO THE OPERATION UNIT.

The ever-growing work of the infirmary has necessitated the increase to the operation unit which is shown on the plan we illustrate to-day.

The block before the alteration comprised an operation theatre and two rooms, one of which was used for anæsthetising, the other for sterilising. The plan shows the walls of the new work blacked in solid, and the walls not disturbed are shown hatched. Access to the theatre block is off the main corridor of the infirmary, which is 10 ft. wide, and is entered through a cut-off lobby, and the corridor leading to the theatres is 8 ft. wide, with additional space near the actual theatres. In the altered block a second theatre has been built, and the size of the old one has been reduced by a few feet, thus giving two theatres each 18 ft. by 17 ft. Large windows and skylights are shown, and have a northern aspect, and in addition three windows on one side. To each theatre a recess for sterilising and washing instruments has been added, and a sink provided. The two old rooms referred to have been thrown into one (marked sisters' room) which is used for preparation and wash-up, and for sterilising dressings, etc. The two steam sterilisers are placed at the end of the room and can be cut off by revolving shutters.

Three new rooms have been added on the other side of the corridor, being, respectively, anæsthetic room, surgeons' room, and bathroom. The provision of the latter should doubtless prove a useful addition. With two theatres being worked from one entrance, there must at times be a danger of overcrowding. This seems to us a grave practical objection to the plan which otherwise has been worked out and considered in detail.

The architects are Messrs. Young and Hall.

## Figures and Tables

**Figure f1:**